# Transcription factor ELK1 accelerates aerobic glycolysis to enhance osteosarcoma chemoresistance through miR-134/PTBP1 signaling cascade

**DOI:** 10.18632/aging.202538

**Published:** 2021-02-17

**Authors:** Qiang Zhang, Jiaqi Wu, Xiangfeng Zhang, Le Cao, Yongping Wu, Xudong Miao

**Affiliations:** 1 Foot and Ankle Group of Department of Orthopaedics, The Second Hospital Affiliated to Zhejiang University School of Medicine, Hangzhou 310000, China; 2 Trauma Group of Orthopaedics, The Second Hospital Affiliated to Zhejiang University School of Medicine, Hangzhou 310000, China

**Keywords:** osteosarcoma, chemoresistance, aerobic glycolysis, ELK1, microRNA-134

## Abstract

Osteosarcoma is a malignancy that primarily affects children and young adults. The poor survival is largely attributed to acquisition of chemoresistance. Thus, the current study aimed to elucidate the role of ELK1/miR-134/PTBP1 signaling cascade in osteosarcoma chemoresistance. Doxorubicin (DXR)-resistant human osteosarcoma cells were initially self-established by continuous exposure of MG-63, U2OS and HOS cells to increasing DXR doses. Osteosarcoma chemoresistance *in vitro* was evaluated using CCK-8 assays and EdU staining. Aerobic glycolysis was evaluated by lactic acid production, glucose consumption, ATP levels, and Western blot analysis of GLUT3, HK2 and PDK1 proteins. The nude mice were injected with 5.0 mg/kg DXR following the subcutaneous transplantation of osteosarcomas. PTBP1 was upregulated in tumor tissues derived from non-responders to DXR treatment and correlated with patient poor survival. PTBP1 enhanced chemoresistance in cultured osteosarcoma cells *in vitro* and *in vivo* by increasing aerobic glycolysis. Additionally, miR-134 inhibited translation of PTBP1. ELK1 bound to miR-134 promoter and inhibited its expression. Overexpressed ELK1 enhanced chemoresistance and increased aerobic glycolysis by downregulating miR-134 and upregulating PTBP1 in DXR-resistant cells. Altogether, the key findings of the present study highlight ELK1/miR-134/PTBP1 signaling cascade as a novel molecular mechanism underlying the acquisition of osteosarcoma chemoresistance.

## INTRODUCTION

Osteosarcoma represents one of the most prevalent bone malignancies among the pediatric and adolescent population, with studies reporting an incidence of 4 million each year and a peak incidence at the age of 15-19 years [1, 2]. Owing to its highly aggressive nature and rapid metastasis, the effectiveness of conventional therapy remains severely limited, often leading to poor patient prognosis with a 5-year event-free survival rate of 14% [3, 4]. At present, chemotherapy represents the most effective approach available and has been well documented to treat osteosarcoma, however patients still suffer treatment failure due to acquired chemoresistance of osteosarcoma cells [5]. Aerobic glycolysis, also known as the Warburg effect, has been implicated in the event of chemoresistance in various cancer cells [6]. Thus, an urgent need exists to identify the underlying molecular mechanisms associated with chemoresistance in order to improve the effectiveness of radiation treatment.

Recent research has highlighted that ELK1 advances the progression of osteosarcoma [7]. ELK1 exhibits distinct DNA binding characteristics related to transcriptional activities, which promotes cell migration [8]. ELK1 displays heightened levels of expression in prostate cancer cells [9]. ELK1 is phosphorylated by activating the MAPK/ERK pathways and translocates to the nucleus, resulting in the activation of downstream targets [10]. Shuang et al. concluded that ELK1 could negatively regulate the expression of miR-134 in chemoresistant ovarian cancer cells [11]. The effects of miR-134 have also been exerted on the progression of osteosarcoma [12]. As a class of small noncoding RNAs, miRNAs have been reported to significantly influence various biological processes, including chemoresistance of cancers, via their target genes [13]. Additionally, miR-134 promotes the chemosensitivity of colorectal cancer (CRC) cells to oxaliplatin (OXA) [14], although there is limited data in regard to the expression of miR-134 in relation to chemoresistance in osteosarcoma cells. In addition, microarray analysis in the present study has proved that polypyrimidine tract-binding protein 1 (PTBP1) is a potential target gene of miR-134. PTBP1 has been implicated in the development and progression of various tumors, including tumorigenesis and chemoresistance of cancer cells [15, 16]. Furthermore, miR-145 has been previously reported to target Warburg effect-related genes such as PTBP1 in the bladder [17]. However, their specific mechanisms in osteosarcoma cells resistant to chemotherapy remain largely unknown. Hence, the current study aims to validate if the aforementioned hypothesis was valid and to further explore the mechanisms by which ELK1 enhances the chemoresistance to osteosarcoma cells through miR-134-targeted PTBP1.

## RESULTS

### Online RNA crosstalk analysis

**Figure 1:**
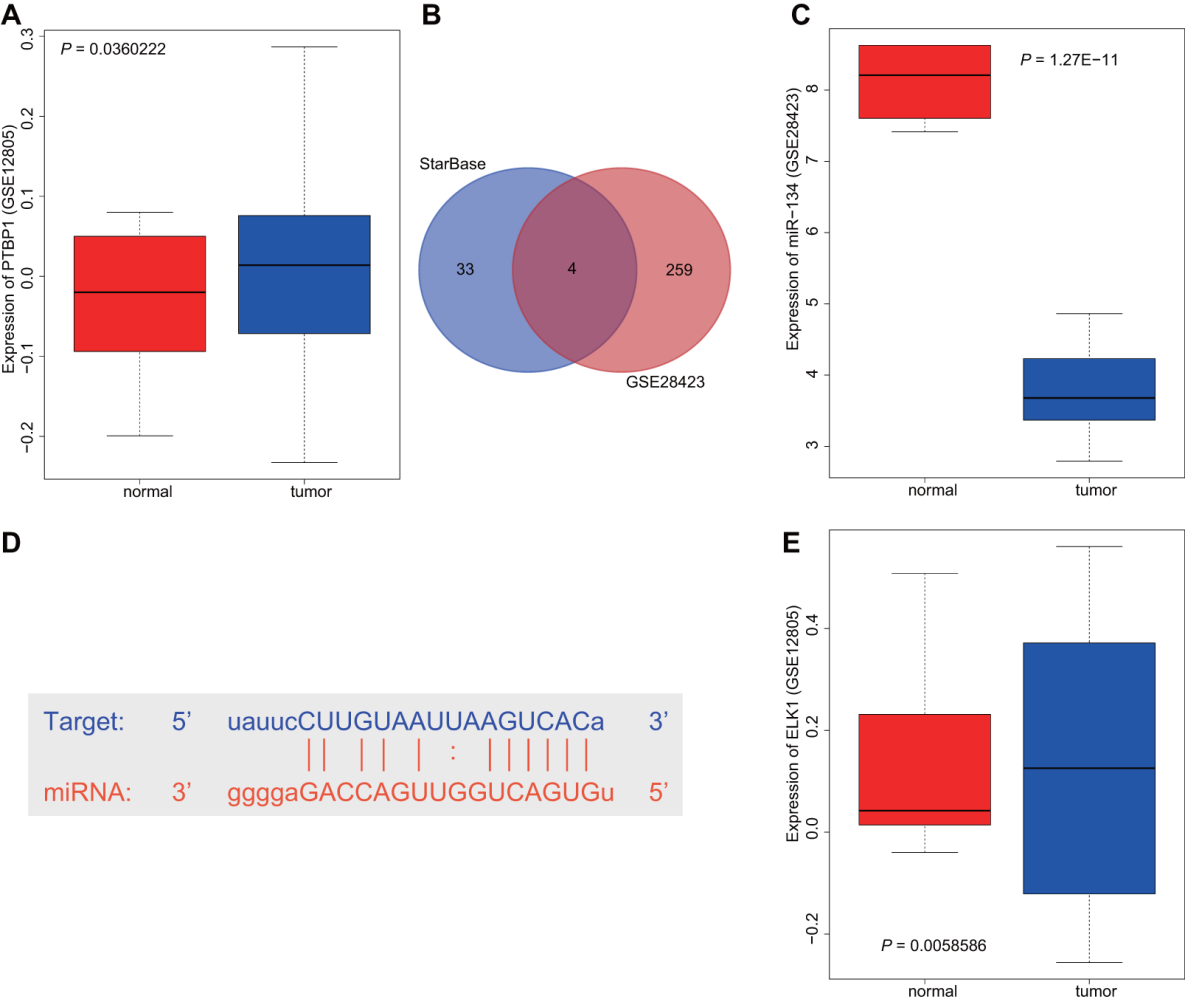
**ELK1 may promote PTBP1 expression by downregulating miR-134 to participate in the chemotherapeutic resistance of osteosarcoma.** (**A**) The boxplot of PTBP1 expression in osteosarcoma-related microarray data GSE12805 from GEO database (https://www.ncbi.nlm.nih.gov/geo/). (**B**) The intersection between the predicted results of miRNAs that regulated PTBP1 and differential analysis in microarray data GSE28423 through StarBase database (https://web.archive.org/web/20110222111721/http://starbase.sysu.edu.cn/). (**C**) The boxplot of miR-134 expression in osteosarcoma-related microarray data GSE12805. (**D**) The binding sites between miR-134 and PTBP1. (**E**) The boxplot of ELK1 expression in osteosarcoma-related microarray data GSE12805.

Differential analysis of the osteosarcoma-related microarray data GSE12805 containing tumor tissues (n = 40) and normal tissues (n = 13) was performed using R language with |log2FC|> 1 and p-value < 0.05 as threshold, with results indicating that PTBP1 was upregulated in osteosarcoma (Figure 1A). Next, the StarBase database was utilized to predict the miRNAs that targeted PTBP1. The intersection between the predicted results and differential analysis for tumor tissues (n = 19) and normal tissues (n = 4) in microarray data GSE28423 were obtained, which revealed four miRNAs (hsa-miR-125a-5p, hsa-miR-139-5p, hsa-miR-134-5p, and hsa-miR-137) (Figure 1B). Through GSE28423 differential analysis, miR-134 was detected to be downregulated in osteosarcoma (Figure 1C). The binding sites of miR-134 and PTBP1 were identified via the StarBase database (Figure 1D). Next, to enhance our understanding of the upstream regulation mechanism of miR-134, differential analysis of GSE12805 was conducted, which revealed that ELK1 was highly expressed in osteosarcoma (Figure 1E). Based on the above findings, we concluded that the transcription factor ELK1 may inhibit PTBP1 expression through miR-134 to promote osteosarcoma chemoresistance.

### Upregulated PTBP1 was associated with DXR resistance and poor survival in osteosarcoma patients

**Figure 2:**
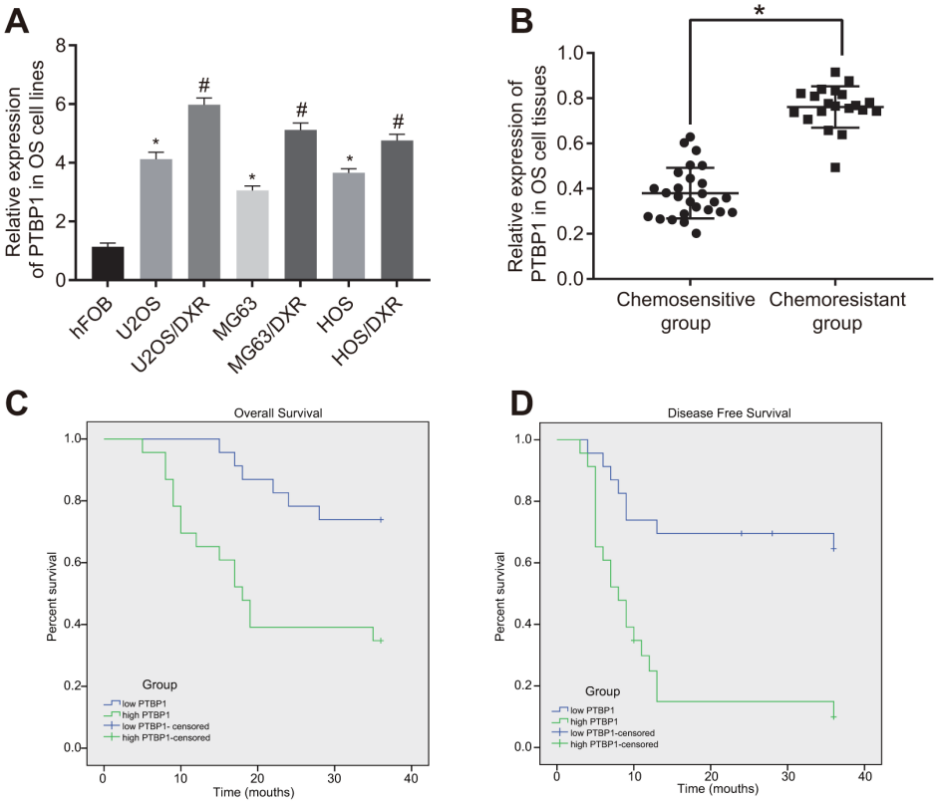
**PTBP1 expression is upregulated in DXR-resistant osteosarcoma tissues and cell lines.** (**A**) PTBP1 expression (normalized to GAPDH) in human osteoblast line, DXR-resistant osteosarcoma cell lines (U2OS/DXR, MG63/DXR, HOS/DXR) and their parental cells determined by RT-qPCR. (**B**) PTBP1 expression (normalized to GAPDH) in tumor tissues derived from non-responders to DXR treatment (n = 24) than that in tumor tissues derived from responders to DXR treatment (n = 22) determined by RT-qPCR, (**C**) Overall survival of osteosarcoma patients with high PTBP1 expression, plotted by Kaplan-Meier method. (**D**) Disease-free survival of osteosarcoma patients with high PTBP1 expression, plotted by Kaplan-Meier method. Values obtained from three independent experiments in triplicate are analyzed by unpaired t test between two groups and by ANOVA followed by Tukey's post hoc test among three or more groups. *p < 0.05 compared with hFOB; *p < 0.05 compared with DXR-resistant osteosarcoma cell lines.

Next, to investigate the relationship between PTBP1 and drug-resistant osteosarcoma cells, we determined the expression of PTBP1 in human osteoblast line (hFOB), DXR-resistant osteosarcoma cell lines (U2OS/DXR, MG63/DXR, HOS/DXR) and their parental cells by RT-qPCR. The results showed that PTBP1 expression in osteosarcoma cell lines was higher than that in hFOB, and it significantly increased in DXR-resistant osteosarcoma cell lines (p < 0.05) (Figure 2A). RT-qPCR results further revealed that PTBP1 expression was higher in tumor tissues derived from non-responders to doxorubicin (DXR) than that in tumor tissues derived from responders (p < 0.05) (Figure 2B). Kaplan-Meier survival analysis demonstrated that patients with higher PTBP1 expression in osteosarcoma tissues had poor overall survival and disease-free survival (Figure 2C, 2D).

### PTBP1 enhanced chemoresistance of osteosarcoma cells to DXR *in vitro*

**Figure 3:**
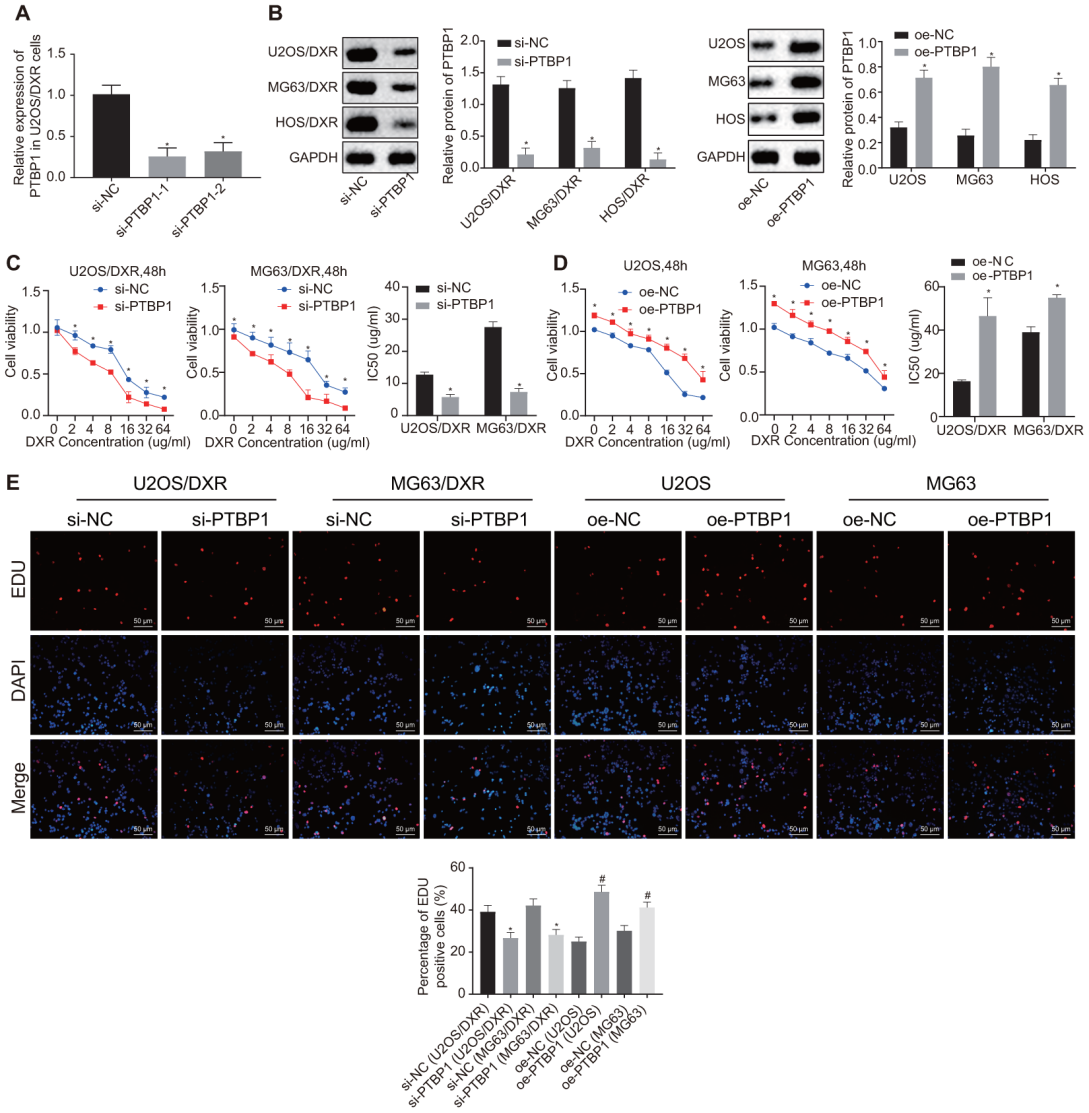
**PTBP1 facilitates chemoresistance of osteosarcoma cells to DXR *in vitro*.** DXR-resistant osteosarcoma cell lines (U2OS/DXR, MG63/DXR, HOS/DXR) were treated with expression vector containing PTBP1 gene and their parental cells with siRNA targeting PTBP1. (**A**) The knockdown efficiency of si-PTBP1 was verified using RT-qPCR in U2OS/DXR cells. (**B**) Western blot analysis determined the protein level of PTBP1 (normalized to GAPDH) in DXR-resistant osteosarcoma cells and their parental cells. CCK-8 assays were performed to examine cell viability and IC50 of DXR in U2OS/DXR cells (**C**) and their parental cells (**D**). (**E**) EdU labeling assays were used to reflect the proliferation of DXR-resistant U2OS and MG63 cells and their parental cells. Values obtained from three independent experiments in triplicate are analyzed by unpaired t test between two groups and by ANOVA with Tukey's test among three or more groups. Values at different time points were tested by repeated measurement ANOVA followed by Bonferroni test. *p < 0.05 compared with parental cells treated with oe-NC or DXR-resistant osteosarcoma cells treated with si-NC.

Following detection of PTBP1 expression in DXR-resistant osteosarcoma tissues and cells, we constructed DXR-resistant osteosarcoma cell lines (U2OS/DXR, MG63/DXR, HOS/DXR) with siRNA targeting PTBP1 and their parental cells (U2OS, MG63, HOS) with expression vectors containing PTBP1 to further explore the effect of PTBP1 on chemoresistance of osteosarcoma cells. RT-qPCR was performed to verify the knockdown efficiency of PTBP1, and PTBP1-1 by siRNA transfection (Figure 3A). Western blot analysis revealed an increased expression of PTBP1 in parental U2OS, MG63, and HOS cells in the presence of PTBP1, and decreased PTBP1 expression in DXR-resistant osteosarcoma cells in the absence of PTBP1 (p < 0.05) (Figure 3B).

After exposure of transfected osteosarcoma cells to DXR at different gradient concentrations (0, 2, 4, 8, 16, 32, and 64 ug/ml), CCK8 assay was employed to detect cell viability and IC50. The results revealed enhanced cell viability and increased IC50 in parental U2OS, MG63, and HOS cells in the presence of PTBP1, while a contrasting trend was identified in the DXR-resistant osteosarcoma cells in the absence of PTBP1 (p < 0.05) (Figure 3C, 3D). EdU assay revealed that cell proliferation was promoted in the parental U2OS, MG63, and HOS cells in the presence of PTBP1, and was inhibited in DXR-resistant osteosarcoma cells in the absence of PTBP1 (p < 0.05) (Figure 3E).

### PTBP1 increased chemoresistance of osteosarcoma cells to DXR by enhancing aerobic glycolysis *in vitro*

**Figure 4:**
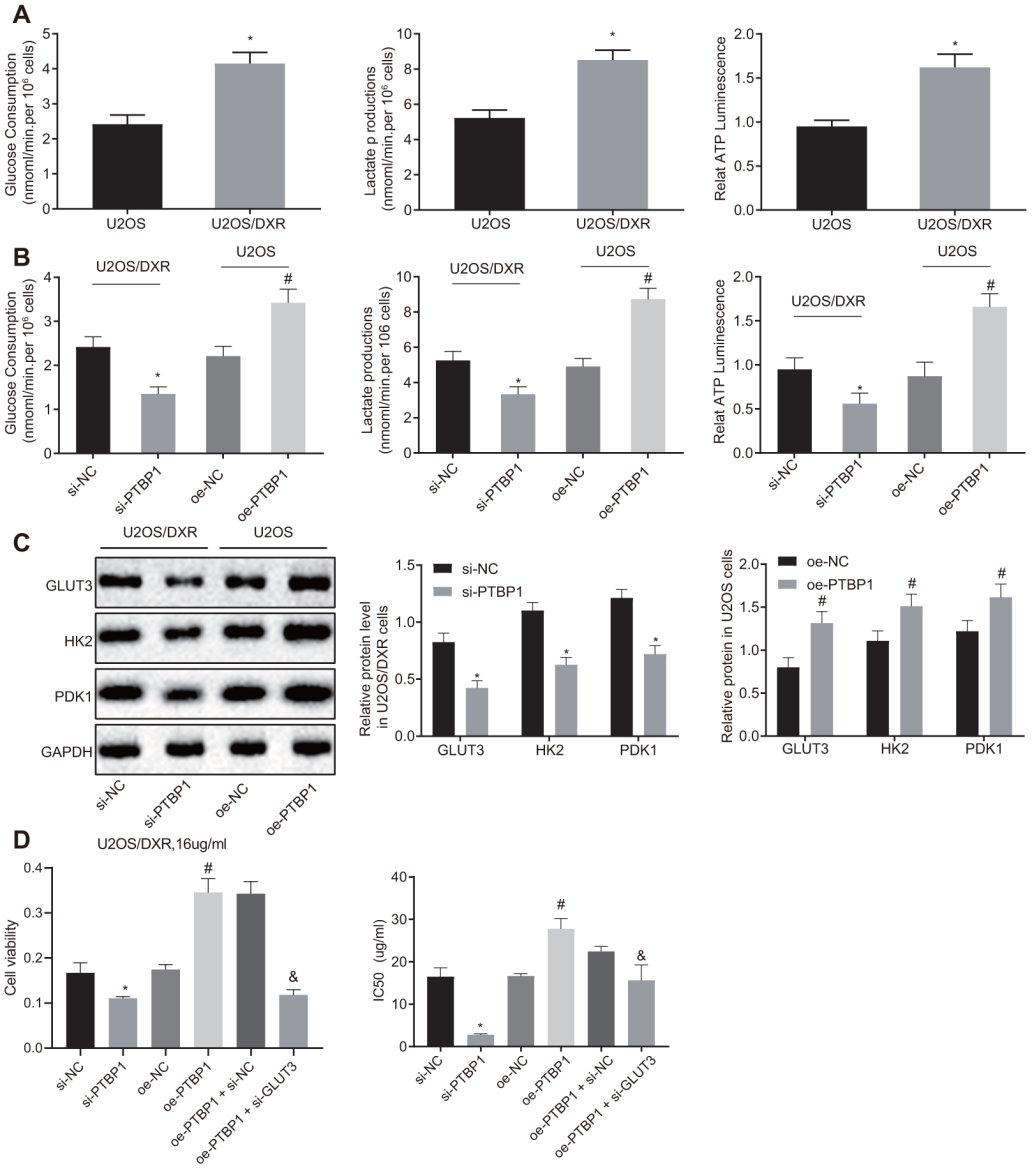
**PTBP1 facilitates chemoresistance of osteosarcoma cells to DXR by enhancing aerobic glycolysis *in vitro*.** U2OS/DXR cells were treated with siRNA targeting PTBP1 and their parental cells were treated with expression vector containing PTBP1 gene. (**A**) The consumption of glucose and lactic acid and the ratio of ATP production in U2OS/DXR cells and their parental U2OS cells. (**B**) The consumption of glucose and lactic acid and the ratio of ATP production in U2OS/DXR cells and their parental U2OS cells. (**C**) Protein levels of GLUT3, HK2, and PDK 1(normalized to GAPDH) in U2OS/DXR cells and their parental U2OS cells. U2OS/DXR cells were treated with siRNA targeting PTBP1, expression vector containing PTBP1 gene and/or siRNA-mediated knockdown of GLUT3, respectively, which were treated with 16 ug/ml DXR. (**D**) Cell viability and IC50 in U2OS/DXR cells. Values obtained from three independent experiments in triplicate are analyzed by unpaired t test between two groups and by ANOVA followed by Tukey's test among three or more groups. *p < 0.05 compared with parental U2OS cells, or U2OS/DXR cells treated with si-NC; # p < 0.05 compared with U2OS cells treated with oe-NC; & p < 0.05 compared with U2OS/DXR cells treated with oe-PTBP1 +si-NC.

Next, to evaluate the relationship between aerobic glycolysis and chemoresistance of osteosarcoma cells, we detected the consumption of glucose and lactic acid, as well as the ratio of ATP production in DXR-resistant U2OS cells and parental U2OS cells, respectively. An increased ratio of glucose and lactic acid consumption and ATP production were detected in the DXR-resistant osteosarcoma cells (p < 0.05) (Figure 4A). The parental U2OS cells were treated with overexpressed PTBP1, while the DXR-resistant U2OS cells were treated with siRNA targeting PTBP1. DXR-resistant U2OS cells in the absence of PTBP1 displayed a decreased ratio of glucose and lactic acid consumption and ATP production (p < 0.05) (Figure 4B), while the parental U2OS cells in the presence of PTBP1 exhibited opposite results.

Western blot analysis results revealed that the expression of aerobic glycolysis-related proteins, GLUT3, HK2, and PDK1, were reduced in DXR-resistant U2OS cells treated with siRNA targeting PTBP1, with the findings displaying a contrasting trend in the parental U2OS cells treated with overexpression PTBP1 plasmids (p < 0.05) (Figure 4C). Next, to further explore the relationship between the enhancement of intracellular aerobic glycolysis induced by elevated expression of PTBP1, U2OS/DXR cells were treated with 16 ug/ml DXR, and then PTBP1 and GLUT3 were overexpressed or silenced, respectively. The CCK8 assay results displayed elevated cell viability and IC50 in U2OS/DXR cells in the presence of PTBP1, and reduced cell viability and IC50 in U2OS/DXR cells in the absence of PTBP1 when compared to their respective NCs. In addition, we identified that siRNA-mediated knockdown of GLUT3 along with overexpressed PTBP1 reversed the effects of overexpressed PTBP1 in U2OS/DXR cells, suggesting that PTBP1 or GLUT3 facilitated chemoresistance of osteosarcoma cells to DXR by enhancing aerobic glycolysis (Figure 4D).

### Silencing of PTBP1 antagonized resistance of osteosarcoma cells to DXR *in vivo*

**Figure 5:**
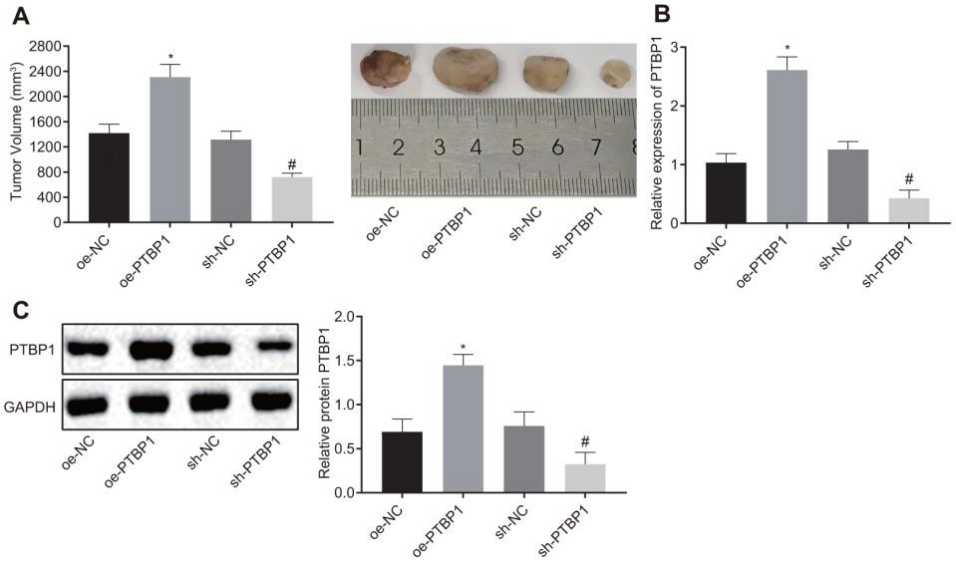
**PTBP1 promotes chemoresistance of osteosarcoma cells to DXR *in vivo*.** Nude mice were were subcutaneously inoculated with PTBP1-overexpressed or PTBP1-depleted U2OS cells. (**A**) Tumor volume in nude mice. (**B**) PTBP1 mRNA level (normalized to GAPDH) in xenograft tumors determined by RT-qPCR. (**C**) PTBP1 protein level (normalized to GAPDH) in xenograft tumors determined by Western blot analysis. Values obtained from three independent experiments in triplicate are analyzed by unpaired t test. *p < 0.05 compared with nude mice injected with si-NC-treated U2OS/DXR cells; # p < 0.05 compared with nude mice injected with oe-NC-treated U2OS/DXR cells.

Next, we set out to further ascertain the role of PTBP1 in enhancing DXR resistant osteosarcoma cells *in vivo*. PTBP1-overexpressed or PTBP1-depleted U2OS/DXR cells were inoculated subcutaneously into nude mice. After the tumors had grown to a size of 50 mm3, the nude mice were treated with DXR, and subsequntly euthanized. The nude mice injected with U2OS cells in the presence of PTBP1 exhibited an elevated tumor volume, while the results in nude mice injected with U2OS cells in the absence of PTBP1 were opposite (p < 0.05) (Figure 5A). RT-qPCR and Western blot analysis results revealed that the expression of PTBP1 was elevated in nude mice injected with PTBP1-overexpressed U2OS cells, and reduced in nude mice injected with PTBP1-depleted U2OS cells (p < 0.05) (Figure 5B, 5C). Altogether, the aforementioned results provided evidence suggesting that the downregulation of PTBP1 suppressed chemoresistance of osteosarcoma cells to DXR *in vivo*.

### ELK1/miR-134/PTBP1 signaling cascade was involved in chemoresistance of osteosarcoma cells to DXR *in vitro*

**Figure 6:**
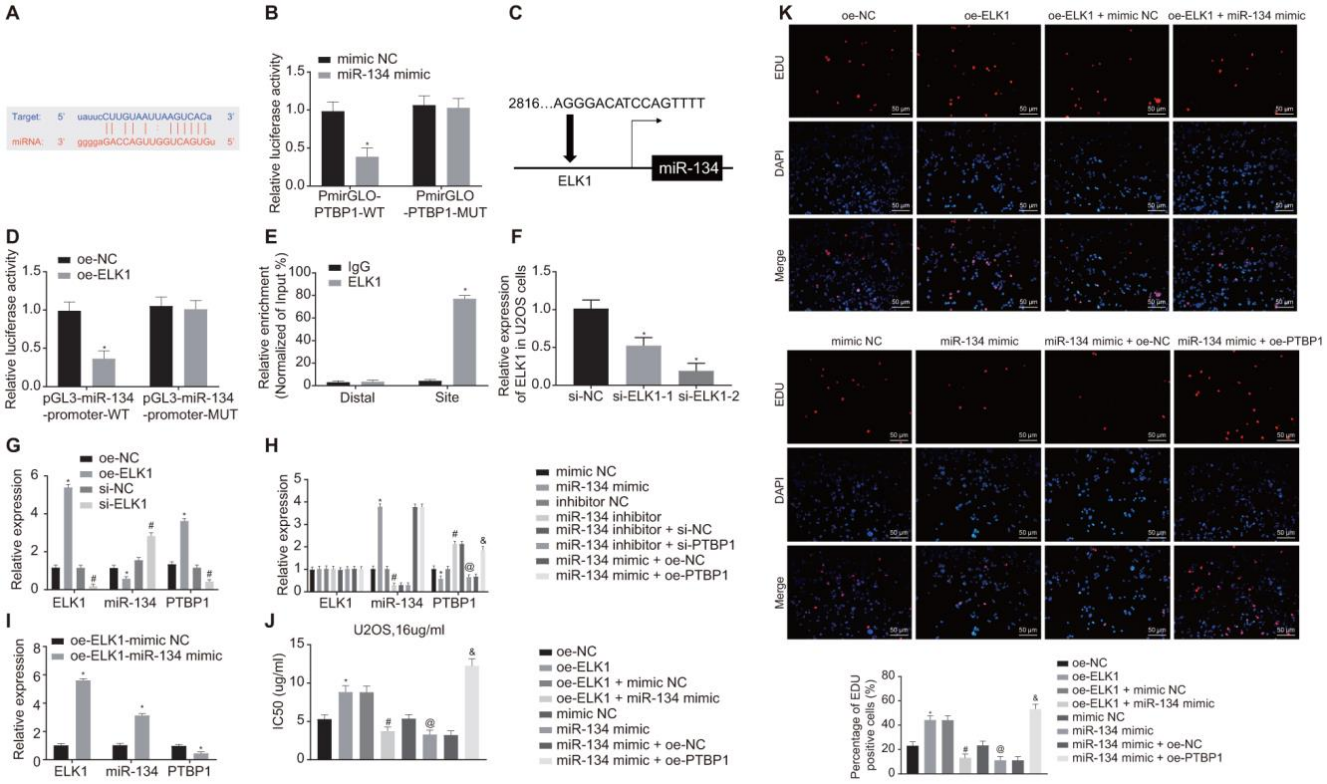
**ELK1 promotes chemoresistance of osteosarcoma cells to DXR by inhibiting miR-134 and elevating PTBP1 expression.** (**A**) Prediction of binding site between miR-134 and PTBP1 using online analysis website microRNA (MicroRNA.org). (**B**) Detection of luciferase activity using dual-luciferase reporter gene assay. (**C**) Prediction of binding site between ELK1 and miR-134 using online analysis website PROMO8.3 (http://alggen.lsi.upc.es/). (**D**) Detection of luciferase activity using dual-luciferase reporter gene assay. (**E**) Intersection role between ELK1 and miR-134 promoter detected by ChIP assay. U2OS cells were treated with expression vectors containing ELK1 gene or si-targeting ELK1 gene. (**F**) The knockdown efficiency of si-ELK1 in U2OS cells verified using RT-qPCR. (**G**) Expression of ELK1, miR-134, and PTBP1 (normalized to GAPDH and U6) in U2OS cells determined using RT-qPCR. PTBP1-overexpressed or -depleted U2OS cells were treated with exogenous miR-134 mimic or miR-134 inhibitor. (**H**) Expression of ELK1, miR-134, and PTBP1 (normalized to GAPDH and U6) in U2OS cells determined using RT-qPCR. (**I**) Expression of ELK1, miR-134, and PTBP1 (normalized to GAPDH and U6) in U2OS cells determined using RT-qPCR. U2OS cells were treated with expression vector containing ELK1 gene, miR-134 mimic, or PTBP1 alone or in combination after DXR treatment. (**J**) Cell viability and IC50 in U2OS cells detected using CCK8. (**K**) Cell proliferation in U2OS cells detected using EdU assay. Values obtained from three independent experiments in triplicate are analyzed by unpaired t test between two groups, by ANOVA followed by Tukey's test among three or more groups, and by repeated measurement ANOVA followed by Bonferroni test at indicated time points. The values at different time points were analyzed by repeated measurement ANOVA, followed by Bonferroni test. In panel C, D, *p < 0.05 U2OS cells treated with oe-NC. In panel F, *p < 0.05 U2OS cells treated with si-NC. In panel G, *p < 0.05 U2OS cells treated with oe-NC; # p < 0.05 U2OS cells treated with si-NC. In panel H, *p < 0.05 U2OS cells treated with mimic NC; # p < 0.05 U2OS cells treated with inhibitor NC; @ p < 0.05 U2OS cells treated with miR-134 inhibitor + si-NC; & p < 0.05 U2OS cells treated with miR-134 mimic + oe-PTBP1. In panel I, *p < 0.05 U2OS cells treated with oe-ELK1.

Next, the online analysis website microRNA predicted that there was a specific binding region between miR-134 and PTBP1 (Figure 6A). Dual-luciferase reporter gene assay verified that miR-134 could target PTBP1 (Figure 6B). The upstream regulatory genes of miR-134 were subsequently subjected to deeper evaluation. The online analysis website PROMO8.3 predicted that there was a specific binding region between miR-134 and ELK1 (Figure 6C). Dual-luciferase reporter gene assay verified that ELK1 could target miR-134 (Figure 6D). ChIP assay also proved the presence of an intersection role between ELK1 and miR-134 promoter (Figure 6E), revealing increased enrichment of ELK1 in the promoter of miR-134.

Next, to further validate the upstream and downstream relationships of ELK1, miR-134 and PTBP1, the U2OS cells were treated with overexpression ELK1 plasmids or silencing ELK1 plasmids. RT-qPCR was then performed to verify the interference efficiency of si-ELK1, and si-ELK-2 was adopted for the following interference experiments (Figure 6F). RT-qPCR results revealed that the expression of miR-134 was diminished while the mRNA levels of ELK1 and PTBP1 increased in the ELK1-overexpressed U2OS cells, while a contrasting trend was identified in ELK1-silenced U2OS cells (p < 0.05) (Figure 6G).

RT-qPCR results revealed reduced PTBP1 mRNA expression, elevated miR-134 expression (p < 0.05), with no significant difference detected in regard to ELK1 mRNA expression in U2OS cells treated with miR-134 mimic (p > 0.05), while a reverse trend was observed in the U2OS cells treated with miR-134 inhibitor (p < 0.05). PTBP1-depleted U2OS cells treated with miR-134 inhibitor showed no obvious difference in expression of ELK1 and miR-134 (p > 0.05), and decreased PTBP1 expression (p < 0.05), and PTBP1-overexpressed U2OS cells treated with miR-134 mimic revealed no obvious difference in expression of ELK1 and miR-134 (p > 0.05), and increased PTBP1 expression (p < 0.05) (Figure 6H) in contrast to their NC. ELK1-overexpressed U2OS cells treated with exogenous miR-134 mimic showed elevated expression of ELK1 and miR-134, and decreased PTBP1 expression (p < 0.05) (Figure 6I).

Next, to detect the effect of ELK1, miR-134, and PTBP1 on DXR-resistance, U2OS cells were treated with oe-ELK1 plasmids, miR-134 mimic, and oe-PTBP1 plasmids alone or in combination after DXR treatment. CCK8 and EdU assay (Figure 6J, 6K) revealed increased IC50 and cell viability in ELK1-overexpressed U2OS cells, which was reversed by further miR-134 overexpression in comparison to U2OS cells treated with NC plasmids (p < 0.05). Compared with the U2OS cells treated with mimic NC, IC50 and cell viability were decreased by miR-134 overexpression, but elevated after overexpression of miR-134 and PTBP1 (p < 0.05). These data confirmed that ELK1 promoted chemoresistance of osteosarcoma cells to DXR by inhibiting miR-134 and upregulating PTBP1 expression.

### ELK1/ promoted aerobic glycolysis to enhance osteosarcoma chemoresistance *in vitro* via miR-134/PTBP1 signaling cascade

**Figure 7:**
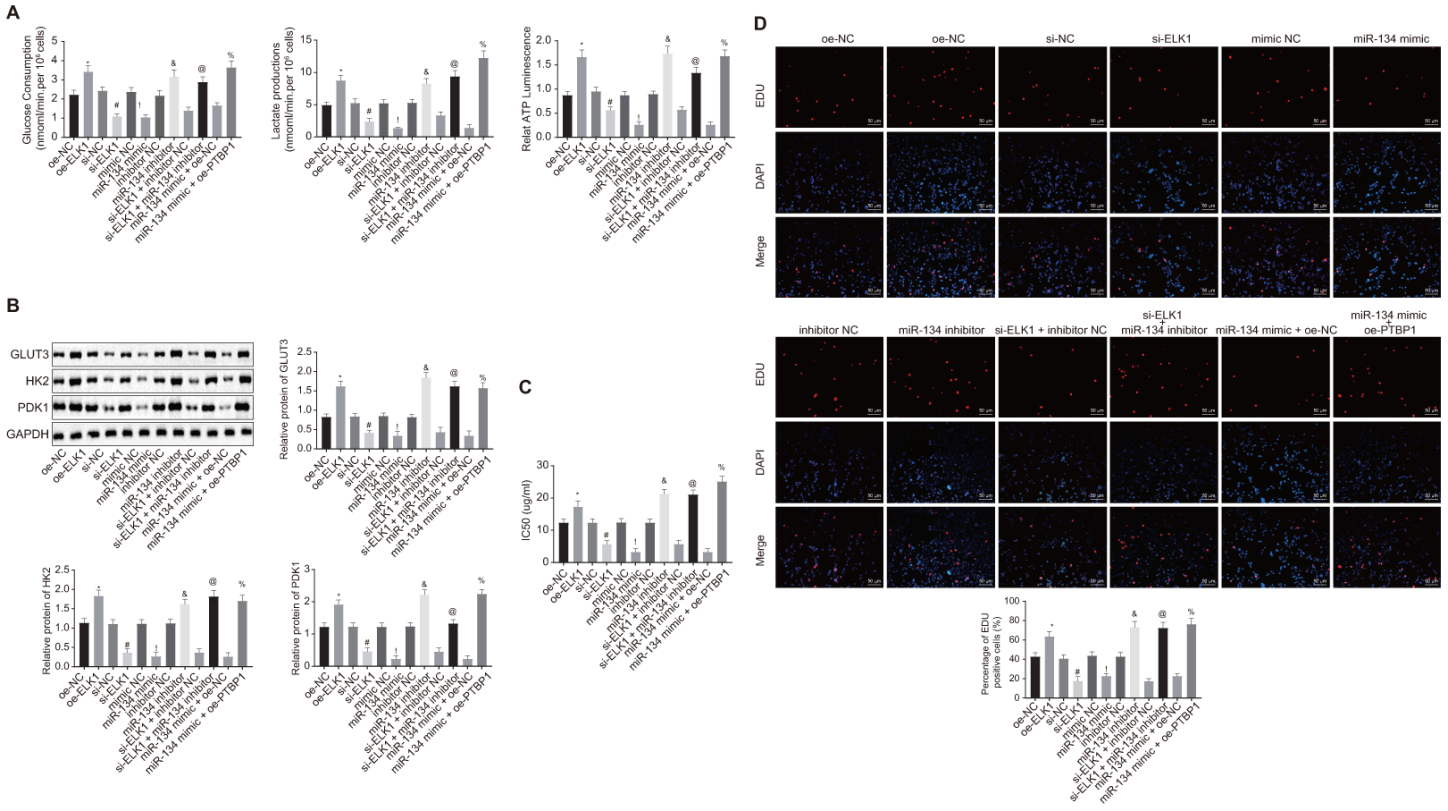
**ELK1 facilitates chemoresistance of osteosarcoma cells to DXR by increasing enhancing aerobic glycolysis through miR-134/PTBP1 axis.** DXR-resistant cells (U2OS/DXR) were treated with expression vector containing PTBP1 gene or siRNA targeting ELK1 gene and/or exogenous miR-134 mimic or miR-134 inhibitor. (**A**) The consumption of glucose and lactic acid and the ratio of ATP production in U2OS/DXR cells. (**B**) Protein levels of GLUT3, HK2, and PDK1 in U2OS/DXR cells. (**C**) IC50 in U2OS/DXR cells detected using CCK8 assay. (**D**) Cell viability in U2OS/DXR cells detected using EdU assay. Values obtained from three independent experiments in triplicate are analyzed by unpaired t test. *p < 0.05 compared with U2OS/DXR cells treated with si-NC; # p < 0.05 compared with U2OS/DXR cells treated with oe-NC; ! p < 0.05 compared with U2OS/DXR cells treated with mimic NC; & p < 0.05 compared with U2OS/DXR cells treated with inhibitor NC; @ p < 0.05 compared with U2OS/DXR cells treated with si-ELK1 + inhibitor NC; % compared with U2OS/DXR cells treated with miR-134 mimic + oe-NC.

In order to determine the roles of ELK1/miR-134/PTBP1 in chemoresistance of osteosarcoma cells after aerobic glycolysis, we detected the consumption of glucose and lactic acid, and the ratio of ATP production in DXR-resistant cells (U2OS/DXR) in the presence or absence of ELK1 and/or miR-134 or miR-134. The findings revealed an elevated ratio of glucose and lactic acid consumption and ATP production in the U2OS/DXR cells in the presence of ELK1 or miR-134 inhibitor, while opposite results were detected in the U2OS/DXR cells in the absence of ELK1 or exogenous miR-134 mimic (p < 0.05). Compared with U2OS/DXR cells treated with si-ELK1 + inhibitor NC plasmids, ELK1-silenced U2OS/DXR cells treated with exogenous miR-134 inhibitor exhibited an elevated ratio of glucose and lactic acid consumption and ATP production, while an opposite trend was observed in the PTBP1-overexpressing U2OS/DXR cells in the presence of miR-134 mimic in comparison to U2OS/DXR cells treated with miR-134 mimic + oe-NC plasmids (p < 0.05) (Figure 7A).

Western blot analysis (Figure 7B) results revealed that the expression of GLUT3, HK2, and PDK1 was elevated in U2OS/DXR cells in the presence of ELK1 or miR-134 inhibitor, but reduced in U2OS/DXR cells in the absence of ELK1 or in the presence of miR-134 mimic (p < 0.05). Compared with U2OS/DXR cells treated with si-ELK1 + inhibitor NC plasmids, ELK1-silenced U2OS/DXR cells treated with exogenous miR-134 inhibitor presented increased expression of GLUT3, HK2, and PDK1, while an opposite trend was identified in PTBP1-overexpressed U2OS/DXR cells treated with exogenous miR-134 mimic in comparison to U2OS/DXR cells treated with miR-134 mimic + oe-NC plasmids (p < 0.05).

The cells in each group were treated with 16 ug/ml DXR. CCK8 and EdU assay (Figure 7C, 7D) results revealed an increase in IC50 and cell viability in U2OS/DXR cells in the presence of ELK1 or miR-134 inhibitor, with contrasting results detected in the U2OS/DXR cells in the absence of ELK1 or in the presence of miR-134 mimic (p < 0.05). Compared with U2OS/DXR cells treated with si-ELK1 + inhibitor NC plasmids, inhibition of miR-34 elevated IC50 and cell viability in ELK1-silenced U2OS/DXR cells, while a reversed trend was observed in the PTBP1-overexpressed U2OS/DXR cells in the presence of miR-134 mimic in comparison to U2OS/DXR cells treated with miR-134 mimic + oe-NC plasmids (p < 0.05).

Altogether, the aforementioned findings demonstrated that ELK1 facilitated the chemoresistance of osteosarcoma cells to DXR by enhancing aerobic glycolysis via the miR-134/PTBP1 axis.

## DISCUSSION

As a primary malignant bone tumor, osteosarcoma is characterized by a highly malignant tendency to rapidly damage the surrounding tissues and to metastasize [18]. Despite the emergence of improved chemotherapy agents against osteosarcoma, resistance to chemotherapy remains a significant stumbling block, resulting in the poor prognosis of patients with osteosarcoma [19]. Thus, our study set out to investigate the role of ELK1 regulating miR-134 and PTBP1 in chemoresistance to osteosarcoma cells. Collectively, our findings demonstrated that ELK1 functioned as a facilitator of chemoresistance in osteosarcoma cells by increasing cell viability and proliferation via miR-134 inhibition and upregulation of PTBP1.

Initially, the data obtained during the present study demonstrated that ELK1 and PTBP1 were highly expressed while miR-134 was poorly expressed in osteosarcoma tissues and cell lines. A recent study suggested that ELK1 expression is elevated in a variety of cancers, including breast cancer and lung non-small cell carcinoma [10]. Previous research has indicated that ELK1 is upregulated in osteosarcoma [20]. Our findings provided evidence confirming that ELK1 could target and downregulate miR-134 expression. ELK1 contributes to the inhibition of miR-134 expression in human cancer [11], which was largely consistent with our findings. The dysregulation of miRNAs is associated with the multiple biological processes, including the chemoresistance of cancer cells [13]. Several miRNAs, such as miR-505 is poorly expressed in osteosarcoma [21]. Interestingly, the expression of miR-134 exhibits a reduction in osteosarcoma [12]. Moreover, a bioinformatics website in combination with dual luciferase reporter gene assay validated that miR-134 could target ELK1, which was negatively regulated by miR-134. A recent study has revealed that PTBP1 is one of the target genes of miR-134 [22]. PTBP1 has been highlighted as an upregulated entity in colon cancer [15], while its increased expression in osteosarcoma is yet to be reported. These finding confirmed that ELK1 and PTBP1 were upregulated and miR-134 was poorly expressed in osteosarcoma tissues and cells.

A notable finding in the present study revealed that ELK1 promoted chemoresistance of osteosarcoma cells to DXR by enhancing aerobic glycolysis and cell viability via inhibition of miR-134 and upregulation of PTBP1. Aberrant glucose metabolism and aerobic glycolysis has been implicated in the chemotherapy resistance of cancer cells by increasing/decreasing cell proliferation and apoptosis [6]. Accumulating evidence has revealed that ELK1, a member of ETS-domain transcription factor family, increases cell growth, differentiation, and survival in tumors, and silencing of ELK1 could suppress cell migration in osteosarcoma cells [7]. A recent study reported that ELK1 is closely related to chemoresistance of cancer cells and depletion of ELK1 could promote the chemosensitivity to CDDP in bladder cancer cells via increment of bladder cancer cell proliferation [23]. Additionally, the downregulation of ELK1 has been shown to promote apoptosis and repress proliferation by increasing miR-134 expression in paclitaxel-resistant ovarian cancer cells [11]. Existing literature has suggested that miR-134 contributes to the elevation of chemosensitivity of CRC tumors to oxaliplatin [14]. Research evidence has indicated that overexpressed miR-134 plays an inhibitory role in xenograft growth and tumor-related angiogenesis in osteosarcoma *in vivo* [12]. Furthermore, the inhibition of PTBP1 by miR-145, inhibits aerobic glycolysis and cell growth in human bladder cancer cells [17]. The suppressed impacts of depleted PTBP1 is also confirmed to inhibit cell growth and proliferation in colon cancer [15]. Taken together, significant evidence exists suggesting that ELK1 is able to promote the chemoresistance of osteosarcoma cells to DXR by promoting aerobic glycolysis and cell viability of osteosarcoma cells via upregulation of miR-134-targeted PTBP1.

**Figure 8:**
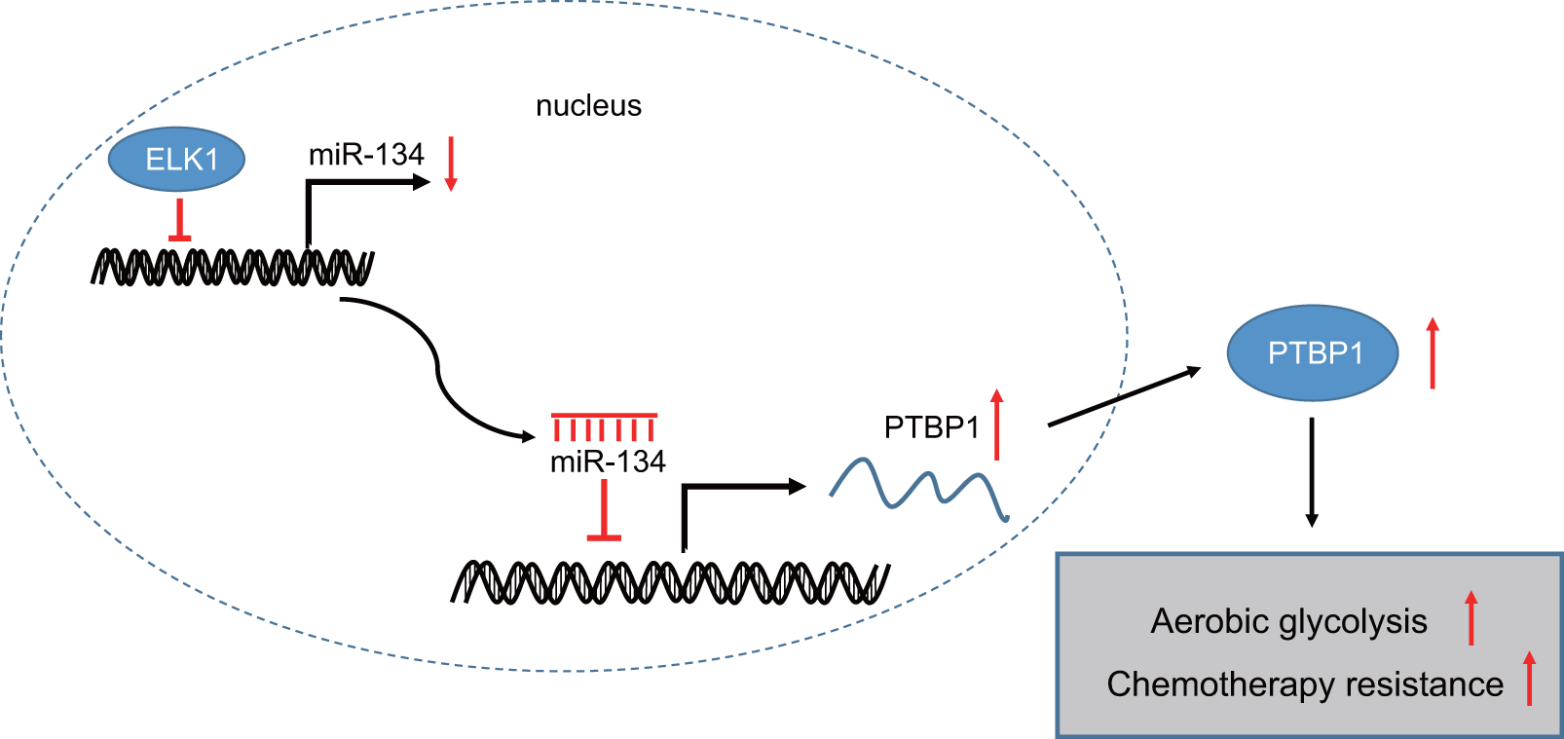
**The mechanism graph of transcription factor ELK1 in chemoresistance in osteosarcoma.** ELK1 promotes aerobic glycolysis by inhibiting miR-134 and upregulating PTBP1 expression, thus enhancing chemotherapeutic resistance in osteosarcoma.

In conclusion, the central findings of the current study present evidence indicating that ELK1 promotes the chemoresistance of osteosarcoma cells to DXR via downregulation of miR-134 and upregulation of PTBP1 (summarized in Figure 8). Therefore, the identification of ELK1 via miR-134 and PTBP1 in chemoresistance of osteosarcoma cells to DXR may aid in facilitating the existing understanding of the mechanisms of osteosarcoma, with potential of serving as a prognostic marker for the chemotherapeutic treatment of osteosarcoma in the future. Future studies are required to fully clarify the specific mechanisms by which ELK1 combined with miR-134 and PTBP1 influence chemoresistance to DXR treatment in osteosarcoma.

## MATERIALS AND METHODS

### Ethics statement

The current study was conducted with the approval of the Ethics Committee of the Second Hospital Affiliated to Zhejiang University School of Medicine (human: 200909028; animal: 201908143). Written informed consent was obtained from all participating patients. All animal experiments were performed in strict accordance with the Guide for the Care and Use of Laboratory Animal by International Committees.

### Study subjects

In this present study, 46 tumor tissue samples were collected from patients diagnosed as primary osteosarcoma (ranging 13 - 42 years old with a mean age of 29.98 ± 8.86 years old) at the Second Hospital Affiliated to Zhejiang University School of Medicine from October 2009 to April 2015. The tissues were divided into doxorubicin (DXR) resistant group (n = 23) and DXR sensitive group (n = 23). Follow-up was conducted for each patient, with April 2018 specified as the deadline.

### Culture of DXR-resistant cell lines

Human osteosarcoma cell lines MG-63, U2OS, and HOS and human osteoblast line (hFOB) from the Institute of Biochemistry and Cell Biology, Chinese Academy of Sciences (Shanghai, China) were cultured in a Dulbecco’s modified Eagle’s medium (DMEM) (Life technologies corporation, Gaithersburg, MD, USA) supplemented with 10% fetal bovine serum (FBS) at 37° C in 5% CO2, penicillin (100 U/ml) and streptomycin (100 μg/ml). DXR-resistant human osteosarcoma cell lines (MG-63/DXR, HOS/DXR and U2OS/DXR) were established by continuous exposure of MG-63, U2OS and HOS cells to DXR. In addition, 1000 ug/mL DXR was added to DXR-resistant cells to maintain DXR-resistant phenotypes.

### Cell treatment

To further ascertain the effect of PTBP1 on the chemotherapy tolerance of osteosarcoma cells, DXR-resistant human osteosarcoma cell lines (MG-63/DXR, HOS/DXR and U2OS/DXR) were transfected with empty vector interference plasmids (si-NC) and PTBP1 interference plasmids (si-PTBP1) respectively. Next, DXR-sensitive osteosarcoma cell lines (MG-63, U2OS and HOS) were transfected with empty vector overexpression plasmids (oe-NC) and PTBP1 overexpression plasmids (oe-PTBP1).

In order to investigate the roles of upregulated PTBP1 expression in the increased intracellular aerobic glycolysis and resistence of osteosarcoma cells to DXR, U2OS/DXR cells were transfected with PTBP1 overexpression plasmids (oe-PTBP1), PTBP1 interference plasmids (si-PTBP1), PTBP1 overexpression plasmid and empty vector silencing plasmids (oe-PTBP1 + si-NC), or PTBP1 overexpression plasmids and GLUT3 silencing plasmids (oe-PTBP1 + si-GLUT3).

Next, to verify the upstream and downstream relationship between ELK1, miR-134 and PTBP1, the U2OS cells were transfected with empty vector interference plasmids (si-NC), empty vector overexpression plasmids (oe-NC), ELK1 overexpression plasmids (oe-ELK1), ELK1 interference plasmids (si-ELK1), empty vector mimic NC plasmids (mimic NC), empty vector inhibitor NC plasmids (inhibitor NC), miR-134 overexpression plasmids (miR-134 mimic), miR-134 interfering plasmids (miR-134 inhibitor), cotransfected with miR-134 interference plasmids and empty vector interference plasmids (miR-134 inhibitor + si-NC), cotransfected with miR-134 overexpression plasmids and empty vector overexpression plasmids (miR-134 inhibitor + oe-NC), cotransfected with miR-134 interfering plasmids and PTBP1 interfering plasmids (miR-134 inhibitor + si-PTBP1), or cotransfected with miR-134 overexpression plasmids and PTBP1 overexpression plasmids (miR-134 mimic + oe-PTBP1).

Further, to detect the effect of DXR resistance of cells, U2OS cells were cotransfected with ELK1 overexpression plasmids and empty vector mimic NC plasmids (oe-ELK1 + mimic NC), ELK1 overexpression plasmids and miR-134 overexpression plasmids (oe-ELK1 + miR-134 mimic), ELK1 interference plasmids and empty vector inhibitor NC plasmids (si-ELK1 + inhibitor NC), or ELK1 interference plasmids and miR-134 interference plasmids (si-ELK1 + miR-134 inhibitor). All plasmids were purchased from Shanghai GenePharma Co., Ltd. (Shanghai, China).

In brief, the cells were seeded into a six-well plate at 10^5^ cells/well. Upon reaching 80% cell confluence, the cells were transfected by lipofectamine 2000 (Invitrogen, Carlsbad, California, USA). And 250 μL serum-free RPMI 1640 medium (Gibco Grand Island, NY, USA) was to dilute 4 μg of target plasmids and 10 μL of Lipo 2000 respectively, and allowed to rest for 5 min. Next, the aforementioned two were mixed, permitted to rest for 20 min, and subsequently added to the culture wells and cultured at 37° C in 5% CO_2_. After 6 h, the medium was replaced with a complete medium and further cultured for 48 h. And then the cells were collected.

### Drug sensitivity tests *in vitro*

After transfection for 48 h in a 96-well plate, fresh medium containing DXR of diffenrent final concentrations (MG63/DXR, HOS/DXR, and U2OS/DXR; 0, 2, 4, 8, 16, 32, and 64 mg/mL) was added to the cells. Three replicates were set for each respective concentration. After incubation for 48 h, cell viability was measured based on the manufacturer's instructions of Cell Counting Kit-8 (CCK-8, Dojindo Molecular Technologies, Inc., Tokyo, Japan).

### RNA isolation and quantitation

Total RNA was extracted using Trizol (15596026, Invitrogen) according to the instructions. Total RNA was reverse-transcribed into cDNA as per the instructions of the reverse transcription reagent kit (RR047A, Takara Bio Inc., Otsu, Shiga, Japan). The reverse transcription quantitative polymerase chain reaction (RT-qPCR) was conducted by ABI7500 quantitative PCR instrument (ABI Company, Oyster Bay, NY, USA) using SYBR Premix EX Taq kit (RR420A, Takara). The primer sequences of LINC00284, NFKB1, MEST, VEGF, CD31, and glyceraldehyde-3-phosphate dehydrogenase (GAPDH) were synthetized by Shanghai Sangon Biotechnology Co. Ltd. (Shanghai, China) (Table 1). GAPDH was regarded as the internal reference for ELK1 and PTBP1, while U6 was considered as the internal reference for miR-134. The expression ratio of target gene between the experimental and control groups was calculated using the 2-ΔΔCt method.

**Table 1:** Primer sequences for RT-qPCR.

**Targets**	**Primer sequence (5’-3’)**
ELK1	F: 5´-CAGCCAGAGGTGTCTGTTACC-3´
	R: 5´-GAG CGCATGTACTCGTTCC-3´
miR-134	F: 5´-UGUGACUGGUUGACCAGAGGGG-3´
	R: 5´-CCUCUGGUCAACCAGUCACAUU-3´
PTBP1	F: 5´-GCGUGAAGAUCCUGUUCAATT-3´
	R: 5´-UUGAACAGGAUCUUCACGCTT-3´
GLUT3	F: 5´-CTTGCTCATTAACAAAAAGGAGGA-3´
	R: 5´-ATCTCCTGGACCACGTCCGAGG-3´
HK2	F: 5´-GGAGGAUGAAGGUAGAAAUTT-3´
	R: 5´-GUCGCUUUGAGACCAAAGATT-3´
PDK1	F: 5´-GCGAATTCATGGCCAGGACCACCAGCCAG-3´
	R: 5´-GCCAGCTGTCACTGCACAGCGGCGTCCGG-3´
U6	F: 5´-GCTTCGGCAGCACATATACTAAAAT-3′
	R: 5´-GGCTTCACGAATTTGCGTGTCAG-3′
GAPDH	F: 5´-GCGUGAAGAUCCUGUUCAATT-3′
	R: 5´-UUGAACAGGAUCUUCACGCTT-3′

F, forward; R, reverse.

### Western blot analysis

The cells were lysed with radio immunoprecipitation assay (RIPA) peptide lysis buffer (R0010, Beijing Solarbio Science and Technology Co. Ltd., Beijing, China) to extract the total protein. The proteins were separated with sodium dodecyl sulfate-polyacrylamide gel electrophoresis (SDS-PAGE) and subsequently transferred onto a polyvinylidene fluoride membrane. The membrane was incubated with rat anti-human primary antibodies PTBP1 (ab133734, 1: 1000, Abcam Inc., Cambridge, UK), GLUT3 (ab41525, 1: 1000, Abcam), HK2 (ab209847, 1: 1000, Abcam), and PDK1 (ab207450, 1: 1000, Abcam) at 4° C overnight. Next, the membrane was washed three times with TBST (5 min per time), followed by incubation with horseradish peroxidase (HRP)-labeled goat anti-rabbit immunoglobulin G (IgG) (1: 20000; ab205718, Abcam) for 1 h. Protein blots were visualized by ECL-associated fluorography (Merck Millipore, Billerica, MA, USA). The data was analyzed using ImageJ 1.48u (National Institutes of Health Inc., USA). The protein content was reflected by the ratio of gray value between proteins and the internal reference (GAPDH).

### Tumorigenicity assay in nude mice

A total of 48 BALB/c nude mice (aging 3 - 5 weeks old and weighing 12 - 18 g) were housed under pathogen-free conditions. Approximately 1.0 × 10^7^ U2OS of cells were suspended in 100 ml PBS and stably transfected with sh-NC (empty silencing plasmids), sh-PTBP1 (PTBP1 silencing plasmids), oe-NC (empty overexpression plasmids), or sh-PTBP1 (PTBP1 silencing plasmids). Next, the stably transfected cells were subcutaneously injected into the right side dorsal region of the nude mice. Tumor growth was measured using vernier calipers once two days. When the tumor was found to have grown to a size of 50 mm^3^, the nude mice were injected with 5.0 mg/kg DXR was administered via the tail vein every two days (three times in total). Four weeks later, all nude mice were euthanized and necropsied. After intraperitoneal injection of 4.0 mg fluorescein (100 mL) (Gold Biotechnology, Inc., St. Louis, MO, USA), all nude mice were photographed under the IVIS@Lumina II system (Caliper Life Sciences, Hopkinton, MA, USA) at a specified time.

### Cell viability assays

The cells were inoculated on the 96-well plates (2.0 × 10^3^ cells/well). The plate with medium and no cells was regarded as the blank control for zero adjustment. After 24 h, each well was incubated added 10 μL CCK8 solution for further incubation at 37° C for 2 h to detect cell viability at 0, 24, 48, and 72 h. The absorbance value was detected at a wavelength of 450 nm using a multifunctional microplate reader (Bio-Rad, Hercules, CA, USA).

### Dual luciferase reporter assays

The promoter region of miR-134 and PTBP1 3'UTR region containing binding sites of wild and mutant reporter plasmids (pGL3-miR-134-promoter-WT, pGL3-miR-134-promoter-MUT, PmirGLO-PTBP1-WT, and PmirGLO-PTBP1-MUT) obtained from Shanghai GenePharma Co. Ltd., were constructed. The PmirGLO-PTBP1-WT and PmirGLO-PTBP1-MUT were co-transfected with miR-134 mimic, and pGL3-miR-134-promoter-WT and pGL3-miR-134-promoter-MUT were co-transfected with OE-ELK into the HEK-293T cells. After transfection for 48 h, cells were collected. Luciferase activity was measured using a luciferase assay kit (D0010, Beijing Solarbio Science and Technology Co. Ltd.). Fluorescence intensity was detected using Promega's GLomax 20/20 Luminometer fluorescence detector (E5311, Shaanxi Zhongmei Biotechnology Co., Ltd., China).

### Chromatin immunoprecipitation (ChIP) assay

The osteosarcoma cells were immobilized with formaldehyde for 10 min to produce DNA-protein crosslinks. Ultrasound (UP-250, Ningbo Scientz Biotechnology Co., LTD., Ningbo, Zhejiang, China) was used to break the cell chromatin into fragments for 15 cycles, 10 s one time at intervals of 10 s. Next, the samples were centrifuged at 12000 rpm for 10 min to collect supernatant, which was later divided into two tubes, and incubated with negative control (NC) rabbit IgG (ab109489, 1: 300, Abcam) and ELK1 antibodies (1: 100, ab32106, Abcam) respectively at 4° C overnight. Protein A Agarose/SaLmon Sperm DNA was added to the cells and centrifuged at 12000 g for 5 min to remove the supernatant. After the non-specific complex had been washed off, the complex was de-crosslinked at 65° C. Phenol/Chloroform was used to extract and purify the recovered DNA fragments. The binding of ELK1 to miR-134 was detected by RT-qPCR with specific primers of miR-134.

### EdU labeling assays

The cells at the logarithmic growth phase were seeded into 96-well plates (2 × 10^3^ - 4 × 10^4^ cells/well) and cultured to normal growth stage. After 24 h, the cells were attached and transfected. Three replicates were set for each group. After transfection for 48 h, the cells were incubated with EdU medium (100 μL/well) for 2 h. Next, the cells were cultured with fixation fluid (100 μL/well) at room temperature for 30 min, incubated with 2 mg/mL glycine (100 μL/well) for 5 min, and subsequently cultured with penetrant (100 μL/well, PBS with 0.5% TritonX-100) for 10 min. Following treatment with 1 × Apollo staining reaction solution without light exposure for 30 min, cells were added with penetrant and incubated with 1 × Hoechst 33342 reaction solution (100 μL/well) for 30 min. After staining, anti-fluorescence quenching tablets (100 μL/well) was added to the cells and photographed under the fluorescence microscope to record the number of Edu-labeled cells. The cells with red nuclei were regarded as the positive labeled cells. The number of positive and negative cells from three random visual fields was counted under the microscope. EdU labeling rate (%) = the number of positive cells / (the number of positive cells + number of negative cells) × 100%.

### Detection of glucose consumption

Cells at the logarithmic growth phase were cultured in flask with phenol red-free RPMI 1640 solution at a concentration of 1.2 × 10^8^ cells/L for 48 h for culture medium collection and used to measure glucose consumption. As per the instructions of the glucose kit (Sigma-Aldrich Chemical Company, St Louis, MO, USA), the 96-well plates were added with the corresponding regeats and incubated at 37° C for 15 min. The absorbance value of each well was measured by automatic enzyme-linked immunosorbent assay system (ELx800, BioTek, USA) at a wavelength of 505 nm. Glucose consumption = (glucose concentration in cell-free medium - glucose concentration in cell medium) × culture medium volume. The relative inhibition rate of glucose consumption (%) = (glucose consumption in cells without DXR - glucose consumption in cells with DXR)/ glucose consumption in cells without DXR × 100%.

### Detection of adenosine triphosphate (ATP)

ATP levels were measured according to the instructions of ATP Level Detection Kit (Promega, Madison, WI, USA). In brief, the cell concentration was adjusted to 3 × 10^5^ cells/mL, and cells were seeded into the 6-well plates (2 mL/well). The blank medium was set as the blank control for zero adjustment. ATP concentration (mmol/grot) = (absorbance value in the experimental group - absorbance value in the control group)/(absorbance value of standard - absorbance value in blank medium) × standard concentration (10^3^ μmoL/L) × sample dilution ratio/total protein concentration (gprot/L).

### Detection of lactic acid production

Lactic acid production was detected based on the instructions of Lactic acid determination kit (BioVision, Milpitas, CA, USA). The test medium was diluted 5, 10 and 20 times respectively, with the ideal value range of A value considered to be between 0.05-0.35. The result obtained from 20 times dilution was adopted. The absorbance value of standards and samples was measured by automatic enzyme-linked immunosorbent assay system at wavelength of 530 nm. Relative inhibition rate of lactic acid production (%) = (lactic acid production in cells without DXR - lactic acid production in cells with DXR) / lactic acid production in cells without DXR × 100%.

### Statistical analysis

All data analyses were conducted using SPSS 21.0 software (IBM Corp., USA). All data were expressed as mean ± standard deviation. Comparisons between two groups were analyzed using an unpaired t test, while comparisons among multiple group were tested by one-way analysis of variance (ANOVA), followed by the application of a Tukey’s test. Comparisons of data were analyzed by repeated measurement ANOVA, followed by Bonferroni test. Patient survival was illustrated using a Kaplan-Meier method and interpreted by Log-rank test. A value of *p* < 0.05 was considered to be reflective of statistical significance.
